# ‘Relative Consent’ or ‘Presumed Consent’? Organ donation attitudes and behaviour

**DOI:** 10.1007/s10198-020-01214-8

**Published:** 2020-07-10

**Authors:** Joan Costa-Font, Caroline Rudisill, Maximilian Salcher-Konrad

**Affiliations:** 1 London School of Economics and Political Science (LSE), Houghton Street, London, WC2A 2AE UK; 2 Arnold School of Public Health, University of South Carolina, Columbia, SC USA

**Keywords:** Organ donation, Relative consent, Family veto, European countries, Presumed consent, I18, Z1

## Abstract

Legislation, in the form of presumed consent, has been argued to boost organ donation but most evidence disregards the practice of seeking relative’s consent, which can either ‘veto’ donation decisions, or ‘legitimize them’, by removing any possible conflict with the donor’s family. We study the effect of presumed consent alongside family consent on individuals’ willingness to donate (WTD) one’s own and relatives’ organs, and on actual organ donation behaviours. Using data from 28 European countries for the period 2002–2010, we found that presumed consent (PC) policies are associated with increased willingness to donate organs, but this effect was attenuated once internal family discussions on organ donation were controlled for. Our findings indicate that relative’s consent acts as a veto of donation intentions and attenuates the effect of regulation on actual donations. More specifically, PC increases WTD one’s own and relatives’ organs in countries where no family consent is required. Consistently, we find that family consent attenuates the influence of regulatory environment on actual donations. The effect is driven by the influence of family discussions which increased WTD, and in combination with presumed consent translated into higher organ donation rates.

**Electronic supplementary material:**

The online version of this article (10.1007/s10198-020-01214-8) contains supplementary material, which is available to authorized users.

## Introduction

Organ supply shortages against increasing demand have resulted in long transplantation waiting lists in the United States (US) and Europe. Evidence shows that in the US, only 42% of eligible organ donors end up actually donating [1]. Similarly, in the European Union (EU), nearly 50,000 patients were waiting for a kidney transplant and over 63,000 were waiting for an organ by the end of 2013. [2] Estimates are that about 4100 patients died while on waiting lists in the EU in 2013. [2] Hence, there is wide scope and need for increasing donation rates.

Although the legislative environment and in particular the effectiveness of presumed consent legislation play a role to incentivize organ donation [3–6], donation decisions are either expressed in individuals’ wills prior to death or—in most cases—posthumously by family members. To date, we know little about the influences of relatives’ decisions about their loved ones’ organs on actual donation rates. One of the major challenges to increasing organ donation rates is that of relatives’ vetoes, often irrespective of the donor decision [7–9]. Unless donation has been discussed with the deceased or has been written in an advanced directive or living will, family members might make choices based on their own opinions, or delay the process, making donation unfeasible. Hence, the family plays a key role in determining the final donation decision [10] and family refusal can impede organ donation [11, 12]. Approximately half of families in the US and 43% in the UK object to donation in comparison to 20% in Spain, a country often touted as having relatively high organ donation rates [13–15].

Previous research using procurement data across 22 countries worldwide indicates that presumed consent policy has an impact on donation rates [3]^.^ It has been found to increase organ donations and willingness to donate organs in Europe [5, 6, 16]^.^ A number of other studies have examined the empirical determinants of effective organ procurement rates and the role of legislation [17–19]. However, a systematic review found that there is more to cross-country differences in donation than the presence of presumed consent legislation [20]. Recent evidence from the introduction of presumed consent in Wales shows a reduction in the number of organ donors [21]. For example, Kessler and Roth [22] document that in making next-of kin decisions, relatives are more likely to support the donation of a relative’s organ if the deceased did not ‘opt in’ rather than when explicitly opted out. Therefore, it remains important to disentangle the effect of family consent from that of legislative environment. To understand the role of family consent to a relative’s donation, it is important to explore the issue of family discussions in organ donation.

This paper examines whether organ donation legislation influences both the individual and relatives’ donation attitudes by vetoing (or delaying) a potentially viable and even intended donation. Prior studies have disregarded this effect, potentially overestimating the positive impact of presumed consent legislation in the presence of family vetoes and limited discussion around donations to make informed decisions. The analysis of attitudes is particularly important in the context of organ donation decisions. Indeed, decisions about organ donation often do not allow much time to make an informed decision, hence pre-approved attitudes might make a difference. In these circumstances, attitudes can be a better proxy of future donations. That said, whether attitudes explain actual donation, and implicitly whether family vetoes donation decisions might depend on the regulatory environment. More specifically, one would expect individual attitudes to donating a relative’s organs to be more important in countries with larger reliance on family consent.

We attempt to answer two questions. First, does the presence of family consent reduce organ donation? Second, does family consent reduce donations more in opt-out rather than in opt-in policy settings? Countries select into opt-in or opt-out policy environments. In the absence of written consent (opt-in), family typically gets involved by default in the organ donation decision in two possible ways. First, if the family member has made no choice and families are allowed to veto opt-in decisions. Second, in some countries such as Germany, family can veto the opt-in decision if they provide good arguments for a change in the person’s mind after the consent statement. Hence, family consent might be an explanation as to why opt-in countries might well observe an attenuated effect on donations, which makes them similar to opt-out countries. More generally, the presence of family vetoes depends on individual family cultural characteristics alongside country specific influences such information system characteristics, hospital processes and family support for the deceased.

Our study focuses on European data. European countries exhibit very significant heterogeneity in organ donation legislation [23], making them specifically suitable for the purposes of our study. In the EU, there are two types of institutional settings for organ donation: informed consent (opt-in) or presumed consent (opt-out). In countries with informed consent or ‘opt-in’ legislation, such as the UK, Germany, and Sweden, an individual or his/her family must give explicit permission for organ removal. Presumed consent countries such as Spain, Portugal, and Austria, assume universal consent without explicit registration otherwise.

We draw upon a large number of attitudinal observations from three waves of Eurobarometer surveys (2002, 2006, 2009), which are representative data from the European Union and extend findings from other studies [4–6]. In total, these provide 51,313 observations. We study the effect of legislative environment alongside family consent as well as known factors from the literature predicting organ donation rates and willingness to donate in addition to country and time fixed effects. The inclusion of time and country-specific effects are our preferred specification as they net out the effect of country specific features (such as culture), or time invariant characteristics (such as the effect of events that are specific to a year). In addition, our specification includes a number of important controls for alternative explanations for organ donation such as education level, occupation, gender and age as well as health expenditure, overall country wealth and overall population education levels.

Our findings indicate that family consent reduced WTD and attenuated the effect of actual donations. The next section describes the data and methods. Section three contains the results and additional analyses and a final section concludes.

## Data

### Willingness to donate (WTD) data

We use Eurobarometer surveys 58.2, 66.2 and 72.3 from 2002, 2006 and 2009, respectively, to create a pooled cross-sectional database of 51,313 individual responses (descriptive statistics for the pooled sample are provided in Table 1). The Eurobarometer surveys are Europe-wide surveys with a representative national sample from each EU country and allow us to form a set of repeated cross sections with observations from 15 countries in 2002 and 28 countries in 2006 and 2009 (current EU countries plus Turkey and United Kingdom, and excluding Malta for which no relevant data were available). Eurobarometer surveys are conducted on a multi-stage random sampling basis. Primary sampling units at the national population level were taken on a random basis according to each country’s distribution of metropolitan, urban and rural residents. In the second stage, a cluster of addresses was randomly selected from each primary sampling unit. Addresses were chosen systematically using standard random route procedures, beginning with an initial address selected at random. Respondents within each household were selected at random for face-to-face interviews. The advantage of the procedure employed was that the response rate effectively becomes 100% because of random sample cluster replacement strategies such that when one respondent opts out, another with similar characteristics is interviewed. Caveats to the survey include sampling procedure methods and difficulties associated with measuring income and education among EU member states [27–29].

**Table 1:** Descriptive statistics of Eurobarometer sample

Variable	2002	2006	2009	Pooled sample
Sample size, *N*	10,675	20,194	20,444	51,313
Willingess to donate own organs, % (SE)	73.5 (0.6)	67.3 (0.5)	64.5 (0.5)	67.4 (0.3)
Willingness to donate family member’s organs, % (SE)	69.5 (0.7)	68.5 (0.5)	65.2 (0.5)	67.3 (0.3)
Have discussed organ donation in family, % (SE)	53.2 (0.7)	44.5 (0.6)	42.3 (0.5)	45.4 (0.3)
Living in a country with presumed consent legislation, % (SE)	57.3 (0.7)	61.8 (0.6)	66.3 (0.5)	62.8 (0.3)
Living in a country with routine family consent, % (SE)	72.3 (0.6)	69.4 (0.5)	73.9 (0.5)	71.8 (0.3)
Married or living together, % (SE)	58.9 (0.7)	61.1 (0.6)	63.6 (0.5)	61.6 (0.4)
Female, % (SE)	50.9 (0.7)	50.9 (0.6)	50.5 (0.5)	50.6 (0.3)
Age, % (SE)				
15–24 years	16.5 (0.6)	15.1 (0.4)	16.2 (0.4)	15.8 (0.3)
25–44 years	36.5 (0.7)	35.7 (0.6)	34.9 (0.5)	35.5 (0.3)
45–64 years	28.8 (0.6)	29.9 (0.5)	30.2 (0.5)	29.8 (0.3)
65 + years	18.2 (0.6)	19.3 (0.4)	18.6 (0.4)	18.8 (0.3)
Education (age when stopped full-time education), % (SE)				
< 16 years	26.2 (0.6)	24.4 (0.5)	26.8 (0.5)	25.7 (0.3)
16–19 years	40.7 (0.7)	41.6 (0.6)	40.7 (0.5)	41.0 (0.3)
20 + years	22.4 (0.6)	24.4 (0.5)	23.7 (0.5)	23.7 (0.3)
Still studying	10.7 (0.5)	9.6 (0.3)	8.8 (0.3)	9.5 (0.2)
Urban residency, % (SE)	38.6 (0.7)	24.9 (0.5)	28.1 (0.5)	29.0 (0.3)
Landline phone ownership, % (SE)	84.3 (0.5)	75.9 (0.5)	69.7 (0.5)	75.0 (0.3)
Mobile phone ownership, % (SE)	73.3 (0.6)	80.6 (0.4)	85.6 (0.4)	81.2 (0.3)
Occupation, % (SE)				
Unemployed	5.4 (0.3)	5.8 (0.3)	6.7 (0.3)	6.1 (0.2)
Self employed	8.4 (0.4)	7.9 (0.3)	7.7 (0.3)	7.9 (0.2)
White collar worker	20.8 (0.6)	22.6 (0.5)	20.1 (0.4)	21.2 (0.3)
Manual worker	21.7 (0.6)	20.4 (0.5)	21.5 (0.5)	21.1 (0.3)
House person	10.9 (0.4)	8.7 (0.3)	11.8 (0.4)	10.4 (0.2)
Retired	22.6 (0.6)	25.0 (0.5)	23.5 (0.4)	23.9 (0.3)
Student	10.2 (0.5)	9.6 (0.3)	8.7 (0.3)	9.3 (0.2)

Respondents were asked about their WTD their own organs as well as those of a relative, whether they have discussed organ donation with their family, and relevant socio-demographic variables. Population size weights were applied to all Eurobarometer data in proportion to the total population of countries included in the sample.

### Actual donation behaviour

We exploit a number of additional data sources to supplement our analysis of the Eurobarometer datasets. Given that actual donation can only be measured at the aggregate level, we combine aggregate country-level donation rates with aggregated data on attitudes towards organ donation from the Eurobarometer surveys. Actual deceased organ donation rates for each country for years 2001–2010 were assembled primarily from the International Registry on Organ Donation and Transplantation [24]. Missing data were supplemented with data from the Global Observatory on Donation and Transplantation. [25] We included the number of deaths from motor vehicle accidents and cerebrovascular causes per 100,000 population [30] and gross domestic product (GDP) per capita^.^ [31] We also included the percentage of residents with tertiary education [32] and a dummy for countries that are predominantly Catholic [33]. Information on the type of organ donation legislation and protocols of seeking family approval came from a variety of sources [3, 6, 20, 34–36].

Table 2 shows descriptive statistics for the aggregate level. Overall, we have a three times larger sample of countries that use presumed consent (PC) than informed consent. The latter were similar in term of income per capita but informed consent countries exhibited higher per capita health expenditure. Cadaveric organ donation rates per million population were higher in presumed consent countries. More countries with presumed consent legislation were predominantly Catholic. Countries adopting an informed consent policy had higher GDP per capita and health expenditure per capita than countries with presumed consent policies. There was no statistically significant difference between the two sets of countries for the number of deaths from motor vehicle accidents and cerebrovascular causes.

**Table 2:** Descriptive statistics of 28 countries in the sample

Variable	Presumed consent	Informed consent	*p* value (two-sample *t* test)
*N*	21	7	
Cadaveric organ donation rate, per million population (SD)	16.2 (8.1)	12.1 (5.2)	< 0.0001
Health expenditure per capita, constant (2005) US$ (SD)	2,033 (1,698)	2,930 (1,906)	0.0003
GDP per capita, constant (2005) US$ (SD)	24,996 (13,818)	28,761 (11,599)	0.0412
Deaths from motor vehicle accidents, per 100,000 population (SD)	16.7 (21.1)	14.7 (24.1)	0.5012
Cerebrovascular deaths, per 100,000 population (SD)	77.2 (50.1)	74.5 (58.7)	0.7133
Catholic countries, *N* (%, SD)	12 (57.1%, 49.6)	2 (28.6%, 45.5)	< 0.0001
Common law countries, *N* (%, SD)	1 (4.8%, 21.3)	2 (28.6%, 45.5)	< 0.0001
Married or living with partner, % (SD)	62.1 (4.7)	59.6 (5.1)	0.0003
Tertiary education attainment among 30–34 years olds, % (SD)	27.2 (10.6)	32.0 (10.2)	0.0012
Population 0–14 years, % (SD)	16.1 (1.9)	17.5 (2.1)	< 0.0001
Population 65 + years, % (SD)	15.9 (2.1)	15.1 (2.3)	0.0076
Informed consent	Denmark, Germany, Ireland, Lithuania, Netherlands, Romania, United Kingdom		
Presumed consent without routine family consent	Austria, Czech Republic, Latvia, Luxembourg, Poland, Sweden		
Presumed and routine family consent	Belgium, Bulgaria, Croatia, Cyprus, Estonia, Finland, France, Greece, Hungary, Italy, Portugal, Slovak Republic, Slovenia, Spain, Turkey		

## Empirical strategy

Our empirical strategy rests on running two sets of empirical specifications to study the effect of family consent alongside of legislative environment. The first refers to examining at the individual level whether individuals’ WTD their own or family members’ organs depend on presumed consent and family consent. This will allow us to understand whether legislation is associated with differences in behaviours. The second set of models comes from an aggregate level specification using actual country level donation rates as the dependent variable. At both the individual and aggregate levels, several models are specified, going from a “naïve” specification that focuses only on the role of organ donation policies to comprehensive, and preferred specifications taking into account how attitudes towards organ donation are discussed within the family.

### Individual-level specifications

We study the association between legislation and individual-level WTD using the standard dependent variables of WTD one’s own organs or WTD those of a relative. Previous studies interpreted WTD as an expression of the intent to pursue a behaviour and identified some dimensions of what actually happens during organ donation decisions. This model would implicitly incorporate specific information such as whether a family discussion had taken place regarding wishes of a family member. However, here, we relax such an assumption by first investigating individuals’ donation attitudes (as a proxy for intended behaviours), followed by an analysis of actual donation rates. In addition to the legislative environment, individuals can express favour towards organ donation as an abstract concept but they may not be reflecting how they feel about actual donation for themselves or a family member and instead are thinking about it in the context of society in general. Such ambivalence might explain some disconnect between WTD and actual donation behaviours when such decisions take place.

Our key variables of interest are organ donation legislation alongside family consent, which varies at the country level. Even though no major legislative changes regarding organ donation occurred in individual countries over the period where Eurobarometer data on WTD are available [3, 37], we rely on policy variations between countries and well as changes in control variables between countries to explore the relationship between policies and WTD own and family members’ organs alongside changes in relevant time varying controls. As an important explanation for the effect of family consent, we include information on whether the individual has discussed organ donation with his/her family and two country-level variables—whether the respondent’s country of residence has presumed consent legislation and whether the respondent’s country of residence routinely seeks family consent. Our main interest is at the interaction of legislation and family consent to test for the presence of a ‘family veto effect’.

All individual specifications include a standard set of control explanations for actual organ donation and intentions from previous studies [3, 5, 6]. More specifically, we include gender, education, age, urban/rural residency, marital status, occupation, and whether the individual has a fixed landline in the household and/or a personal mobile phone to control for demographic and socioeconomic characteristics that were associated with altruistic behaviours in the literature.

The empirical specification predicting the probability of an individual’s WTD ($${y}_{ij})$$ is reproduced below as:

1 $${\text{WTD}}\left( {y_{ij} = 1 , \left\{ {Z_{jt} ,X_{it} ,{\text{PC}}_{j} , {\text{FC}}_{j} } \right\}} \right) = Z_{jt} \gamma + X_{ijt} \beta + PC_{j} \gamma + FC_{t} \delta +\varepsilon_{ijt} ,$$

where $Z_{jt}$ reflects country specific covariates, $X_{ijt}$ refers to individual covariates (e.g., whether the individual has discussed donation in the family), ${\text{PC}}_{j}$ stands for presumed consent regulation (specific to country *j*, and in the absence of legislation changes fixed over time and fixed for individuals within the same country), and ${\text{FC}}_{j}$ refers to family consent (again a country-level dummy which does not vary over time, but can vary with respect to presumed consent legislation). We allow for a battery of effects and consider different specifications which vary the number of control variables as well as country and year fixed effects. The latter is our preferred specification as it controls for country-specific variation in culture and time-specific events.

We estimate pooled fixed effects OLS and probit specifications with clustered standard errors to account for correlation within countries and gender. We allow for both country fixed effects to capture the effect of unobservables that are country specific as well as year fixed effects to control for those variables that simply correlate with time, namely time varying unobserved heterogeneity during the period examined that could have influenced our estimates. Hence, our estimates of the role of family vetoes are net of the effects of other potential time and country-specific effects.

We have run a number of checks to test the robustness of our results to different specifications, including probit and logit specifications. In our primary analysis, we had excluded respondents with ‘don’t know’ answers and robustness checks were carried out to examine the sensitivity to such exclusions. When confronted with a decision to donate family members’ organs, respondents who have no formed opinion may be more likely to default to a negative answer. We, therefore, conducted a robustness check of our findings by recoding ‘don’t know’ answers to ‘not WTD’.

Attitudes in the population and policies could impact each other, which poses a major challenge to the estimation of an effect in any one direction. In the absence of a quasi-natural experiment (introduction of a new policy) in the observation period, we investigated the use of instrumental variables to address potential endogeneity. However, we could not identify a strong instrument that could credibly disentangle the effect of national policies on attitudes in the population from a potential reverse effect of attitudes on policies. Rather than using a weak instrument, we elected to continue with a non-instrumented set of specifications [38].

### Actual donations

For the second set of models (aggregate level), we use the log of cadaveric organ donation rates in all countries from our sample from 2001 through 2010 as our dependent variable. We include actual organ donation data from 28 countries over 10 years. There were missing donation rates in 2001 for Estonia, Greece, Lithuania; in 2003, for Luxembourg; and in 2001–2003 for Bulgaria. We include two sets of covariates of interest; institutional and behavioural variables. The institutional variables included organ donation legislation and whether family consent is routinely sought. For the behavioural variable, we include a dummy variable for whether more than 50% of the population in that country had discussed organ donation with their family members. Data for this variable came from the Eurobarometer surveys. Data on organ donation discussion in the family were available for 15 countries before 2006, 27 countries before 2009, and 28 countries for 2009 and 2010. Missing data on attitudes before 2006 are due to countries joining the EU in the 2004 expansion not having been included in previous waves of the Eurobarometer survey. Adding in missing data on actual donation rates leaves a sample of 210 observations for the analysis of the relationship between family attitudes and organ donation rates. We also estimated interaction terms between the behavioural and institutional variables.

Standard control variables include GDP per capita, educational attainment, whether the country is pre-dominantly Catholic and whether it practices common law. We also controlled for deceased organ donor supply by including the number of deaths from motor vehicle accidents and cerebrovascular deaths per 100,000 population. We have followed the analysis of Abadie and Gay [3] in using the log of vehicle accident/cerebrovascular deaths and inclusion of a dummy for common law countries. We did not include health expenditure per capita as it was highly correlated with GDP per capita.

Log annual donation rates in each country ${(y}_{jt})$ are specified as follows:

2 $${\log}(y_{jt} ) = Z_{jt} \gamma + F_{jt} \beta + R_{j} \delta + T_{t} \theta + \varepsilon_{jt} ,$$

where ${Z}_{jt}$ are a set of country-level covariates (eg. educational attainment, GDP per capita); ${F}_{jt}$ is the country-level family covariate (behavioral variable); ${R}_{j}$ are a set of country-level, time-invariant regulatory covariates (institutional variables), including presumed consent legislation and routine family consent; and ${T}_{t}$ are year fixed effects. Given that policy variables do not vary within the period of our study, our preferred specification is a pooled cross-section with both country and time effects with bootstrapped standard errors. Alternative specifications produce comparable results.

We first specify a set of two models to analyse the relationship between legislation and actual donation rates, controlling for a set of country-level covariates and including country effects to control for cultural differences in our set of diverse countries. We then add the behavioural variable (majority of population discussed organ donation) in a second set of models and interact this with organ donation legislation to explain differences in donation rates in our sample, controlling for the same set of country covariates. Finally, we consider the inclusion of country fixed effects to wipe out the effect of country invariant unobservables. We use bootstrapped standard errors in all models (300 iterations).

Our analysis is primarily focused on the set of (at the time of data collection) 28 European countries plus Turkey, for which we conducted regression analyses at the individual level. We test the robustness of our findings using alternative samples (results provided in Table S2). We replicate the results of Abadie and Gay using a sample similar to theirs (excluding the United States, Canada, Australia, New Zealand, for which we have no data). For this sample (the “Abadie and Gay sample”), we exclude from our initial sample Bulgaria, Croatia, Cyprus, Estonia, Greece, Latvia, Lithuania, Luxembourg, Romania, Slovak Republic, and Turkey. We also define a “Western Christian sample” that follows the reasoning of Abadie and Gay [3] for excluding some of the countries in their sample (that are not Western Christian countries). Differently to them, we keep many Eastern European countries in the sample, because data that were not available when Abadie and Gay [3] conducted their study have become available in the meantime. For the “Western Christian sample”, we exclude Bulgaria, Cyprus, Greece, Romania, and Turkey from the full set of 28 countries. Finally, we show that our results hold even when we use a more restrictive sample by limiting the analysis to only 1 year (2010, the last year for which we have data).

## Results

In the pooled sample of all three Eurobarometer waves, we document that 67.4% were WTD their own organs and 67.3% were WTD a family member’s organs (Table 1). There was some variation in attitudes over time, pointing towards less favourable attitudes towards organ donation in 2009 compared to 2002, which could partly be explained by the accession of new EU member states in 2005. In countries with presumed consent, but no practice to routinely seek family consent, WTD decreased between 2002 and 2009 (Figure S1). Average WTD one’s own and a relative’s organs was highest in the group of countries with both presumed consent legislation and family consent policy compared to other groups in 2002 and 2006, but was lower compared to informed consent countries in 2009.

### Family consent and WTD one’s own and a relative’s organs

Our regression estimates predicting individuals’ donation intentions (WTD one’s own and a relative’s organs) show a positive association of presumed consent as compared to informed consent (Table 3). However, this relationship is *only true in countries where no family consent* is required (our preferred specification shows an 15 percentage point (pp) increase in the WTD one’s own organs and 18 pp increase in the WTD family members’), whilst it becomes non-statistically significant when family consent is required. Furthermore, family discussion exerts a large independent association with an individual’s WTD one’s own and relatives’ organs (27 pp increase).

**Table 3:** Estimates for the probability of being willing to donate (WTD) ones own and their relatives organs (28 countries)

Variables	WTD own organs			WTD family member’s organs		
	(3.1)	(3.2)	(3.3)	(3.4)	(3.5)	(3.6)
**Regulation covariates**						
*Donation policy (reference category: informed consent)*						
Presumed consent, no family consent	0.175** (0.077)	0.153** (0.075)	0.154** (0.074)	0.206** (0.102)	0.178* (0.099)	0.179* (0.098)
Presumed and family consent	0.078 (0.055)	0.074 (0.050)	0.076 (0.051)	0.056 (0.069)	0.050 (0.064)	0.049 (0.064)
**Family covariates**						
Organ donation discussed		0.267*** (0.010)	0.274*** (0.015)		0.241*** (0.009)	0.238*** (0.011)
**Interaction regulation/family covariates**						
Interaction organ donation discussed × presumed consent (no family consent)			− 0.007 (0.029)			− 0.004 (0.024)
Interaction organ donation discussed × presumed consent × family consent			− 0.012 (0.023)			0.007 (0.022)
Controls	Yes	Yes	Yes	Yes	Yes	Yes
Country-wave fixed effects	Yes	Yes	Yes	Yes	Yes	Yes
***Model statistics***						
Observations	49,936	49,718	49,718	49,936	49,718	49,718
Pseudo *R*-squared	0.087	0.156	0.156	0.073	0.127	0.127

This specification refers to the probability to donate ones organs and those of relatives. Coefficients for probit specifications are marginal effects. Controls include: married or living together; gender; education (four categories); age group (four categories); urban residency; landline phone ownership; mobile phone ownership; occupation (seven categories); and a range of country-level controls: log GDP per capita; tertiary education attainment; Catholic country; common law country; and log motor vehicle and CV deaths

***Significant at the 1% level

**5% level. SE shown in parentheses

**Fig. 1:**
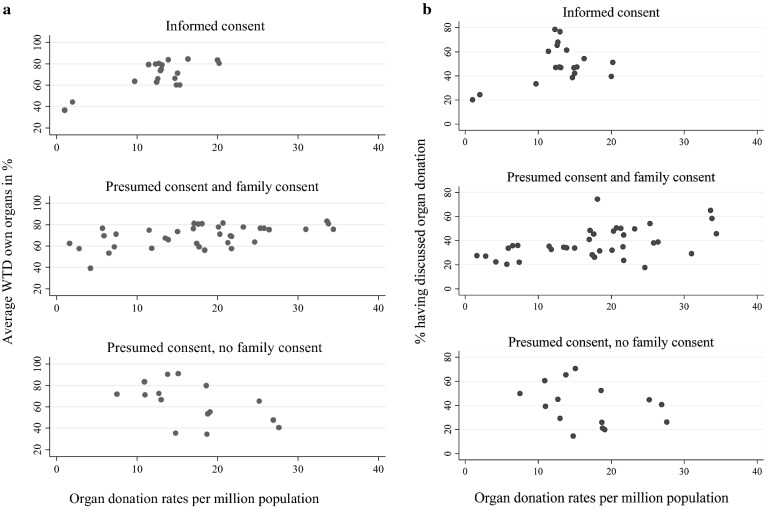
**a** Relationship between average willingness to donate own organs and actual cadaveric donation rates per million population, grouped by organ donation policies (2002, 2006, 2009). Informed consent countries Denmark, Germany, Ireland, Lithuania, Netherlands, Romania, United Kingdom. Presumed and family consent: Belgium, Bulgaria, Croatia, Cyprus, Estonia, Finland, France, Greece, Hungary, Italy, Portugal, Slovak Republic, Slovenia, Spain, Turkey. Presumed consent without family consent countries: Austria, Czech Republic, Latvia, Luxembourg, Poland, Sweden. Values for 2002 only include 15 EU member countries before 2004. **b** Relationship between proportion of the population discussing organ donation and actual cadaveric donation rates per million population, grouped by organ donation policies (2002, 2006, 2009). Informed consent countries Denmark, Germany, Ireland, Lithuania, Netherlands, Romania, United Kingdom. Presumed and family consent: Belgium, Bulgaria, Croatia, Cyprus, Estonia, Finland, France, Greece, Hungary, Italy, Portugal, Slovak Republic, Slovenia, Spain, Turkey. Presumed consent without family consent countries: Austria, Czech Republic, Latvia, Luxembourg, Poland, Sweden. Values for 2002 only include 15 EU member countries before 2004. Source: Data on discussion about organ donation come from Eurobarometer 58.2, 66.2 and 72.3 from 2002, 2006 and 2009. Actual cadaveric donation rates come IRODaT [24], GODT [25]. Source: Willingness to donate organs come from Eurobarometer 58.2, 66.2 and 72.3 from 2002, 2006 and 2009. Actual cadaveric donation rates come IRODaT [24], GODT [25]

Nonetheless, as already reported, there are good reasons to believe that intentions to donate (WTD estimates) does not necessarily translate into actual organ donation behaviour, and more specifically, the regulatory environment and family consent might interfere in such an association. Indeed, Fig. 1a shows a *positive association between the average WTD organs and cadaveric organ donation rates in countries that are subject to either informed consent or presumed consent and family consent*. However, the association turns negative for countries with pure presumed consent policies (without family consent). The overall high rates of WTD in presumed consent countries are at odds with a pattern of consistently lower actual organ donation rates compared to countries with presumed consent (Table 2).

**Fig. 2:**
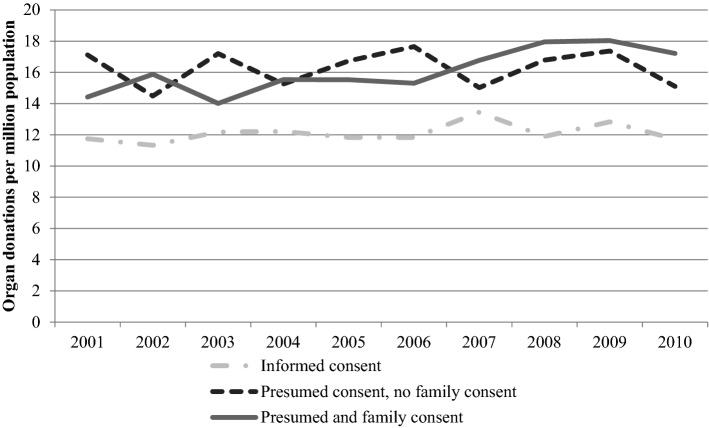
Deceased organ donation rates 2001–2010 in countries without presumed consent; countries with presumed consent only; and countries with presumed consent and routine family consent. Informed consent countries Denmark, Germany, Ireland, Lithuania, Netherlands, Romania, United Kingdom. Presumed and family consent: Belgium, Bulgaria, Croatia, Cyprus, Estonia, Finland, France, Greece, Hungary, Italy, Portugal, Slovak Republic, Slovenia, Spain, Turkey. Presumed consent without family consent countries: Austria, Czech Republic, Latvia, Luxembourg, Poland, Sweden. Values for 2002 only include 15 EU member countries before 2004. Source: IRODaT [24], GODT [25]

Next, Fig. 1b reveals that part of such effects is confirmed when one examines the association between family discussions and actual donation rate. Indeed, a positive association is found between donation rates and family discussions in countries with either informed consent or presumed consent with family consent required. In contrast, no such positive association is found in countries subject to presumed consent with no family consent. Figure 2 shows a time invariant trend that informed consent countries exhibit 20–30% lower organ donation rates. However, among presumed consent countries, those adopting a practice of routinely seeking family approval had higher donation rates than countries with pure presumed consent legislation from 2007 onwards.

### Family consent and actual donation

Next, we proceed with examining the effect of legislative environment and family consent on actual donation. Table 4 shows the coefficients of the regression analysis predicting actual donation rate. We show a significant effect of informed and presumed consent legislation only in naïve models (Table 4) where *the effect of family consent attenuates the influence of regulatory environment*. More specifically, the effect of regulatory environment suggests higher donation rates in presumed consent countries, yet, the presence of family consent attenuates the association of presumed consent by 23% in our preferred specification.

**Table 4:** Estimates for log of cadaveric donation rates, 2001–2010 (28 countries)

Variables	(4.1)	(4.2)
**Regulation covariates**		
*Donation policy (reference category: informed consent)*		
Presumed consent, no family consent	0.484*** (0.142)	1.536** (0.752)
Presumed and family consent	0.401*** (0.122)	1.176*** (0.361)
**Controls**		
Log GDP per capita	0.198 (0.164)	0.681** (0.275)
Tertiary education attainment	0.003 (0.00499)	− 0.001 (0.0107)
Catholic country	− 0.489** (0.219)	0.280 (0.272)
Common law	0.397*** (0.0906)	0.664*** (0.181)
Log motor vehicle and CV deaths	0.005 (0.116)	1.593** (0.707)
Year fixed effects	Yes	Yes
Country effects		Yes
Observations	260	260
*R*-squared	0.410	0.883

This table provides the estimates of regulation covariates on organ donation rates (log transformed) alongside a number of controls, time and country fixed effects. All models include year fixed effects. Standard errors are bootstrapped

***Significant at the 1% level

**5% level

### Channel: family discussion

Nonetheless, such an association is confounded by the extent of family discussion, which is more common when family consent is required or in informed consent countries as suggested by Fig. 1b. Hence, we included in our specification a variable capturing whether the majority in a country had discussed organ donation, which is then interacted with regulatory environments. This variable is retrieved form Eurobarometer surveys and is only available for the countries included in such surveys, hence, our sample is slightly smaller for these analyses. Table 5 reports estimates suggesting that regulatory variables are statistically insignificant when controlling for family discussions. However, once country fixed effects (and hence potential country-specific unobservables) are controlled for (in the preferred model 5.3), we observe a reduction in organ donation rates by 32 pp in countries where the majority had discussed organ donation in their families. However, in countries with presumed consent legislation and a routine policy to seek family consent, this effect is reversed and we observe a 33 pp increase in organ donation rates for countries where the majority of families had discussed organ donation. These findings are robust across all three samples in our robustness checks, and are robust in terms of direction of effect and effect size when restricting the sample to 1 year only (Table S2).

**Table 5:** Estimates for log of cadaveric donation rates, 2001–2010 (28 countries)

Variables	(5.1)	(5.2)	(5.3)
**Regulation covariates**			
*Donation policy (reference category: informed consent)*			
Presumed consent, no family consent	0.0987 (0.117)	0.154 (0.201)	0.433 (1.245)
Presumed and family consent	0.163 (0.103)	0.134 (0.132)	0.369 (0.410)
**Family covariates**			
Organ donation discussed by > 50% of population	0.0440 (0.0957)	− 0.00840 (0.112)	− 0.317** (0.159)
**Interaction regulation/family covariates**			
Presumed consent, no family consent × majority discussed organ donation		− 0.186 (0.230)	0.622 (0.591)
Presumed and family consent × majority discussed organ donation		0.194 (0.139)	0.331** (0.161)
**Controls**			
Log GDP per capita	− 0.126 (0.124)	− 0.103 (0.191)	0.337 (0.548)
Tertiary education attainment	− 0.00238 (0.00488)	− 0.000975 (0.00477)	0.00825 (0.0125)
Catholic country	0.448*** (0.0799)	0.414*** (0.0829)	1.094** (0.542)
Common law	− 0.0344 (0.134)	− 0.0467 (0.112)	0.839 (1.254)
Log motor vehicle and CV deaths	− 0.874*** (0.209)	− 0.845*** (0.221)	− 0.268 (0.427)
Year fixed effects	Yes	Yes	Yes
Country effects			Yes
Observations	210	210	210
*R*-squared	0.426	0.434	0.869

This table provides the estimates of regulation covariates on organ donation rates (log transformed) alongside a number of controls, time and country fixed effects. All models include year fixed effects. Standard errors are bootstrapped

***Significant at the 1% level

**5% level

## Additional analyses

### Robustness checks on individual level models

Table S1 presents the results of robustness checks run for the most complete model (model 3.3), showing that the results do not change materially when using a logit (instead of probit) model. When including don’t know answers (recoded as ‘not willing to donate’, instead of excluding these responses), legislation variables are statistically significant in addition to the family discussion variable.

### Robustness checks on aggregate donation rates

We explored the effect of different subsamples (Table S2) and find results that are almost identical with those in Table 5. The only material difference found in these robustness checks is that statistical significance disappears when restricting the analysis to only 1 year. However, effect direction and sizes still remains consistent with our overall findings.

## Conclusion and policy implications

Using European data, this paper has documented that family consent legislation plays a central role in influencing both organ donation intentions and actual donation rates. Our findings indicate that a policy to routinely seek family consent reduced WTD, and the effect of presumed consent legislation on actual donations was also reduced by 23% when examining family consent. These effects are robust and can be explained by the influence of family discussions. These results suggest that presumed consent by itself often will be inconsistent with a person's desires, because it is rare that people will explicitly opt out of an organ donation policy, as the alternative raises ethical concerns. Hence, a more nuanced policy using an active or even enhanced active choice model, where people are asked to say one way or another as to whether they want to donate (yes or no) (active choice) and giving consequences of what they might lose by not opting in (enhanced active choice), might be a better compromise between opt in vs. out [39].

Other policy approaches that might arise from these findings are removing the idea of family consent particularly in presumed consent environments, although this is unlikely to be feasible from political and societal acceptability perspectives. Ethically, it is a challenge to suggest that the wishes of bereaved relatives be ignored when their loved one has not ‘opted out’ in a presumed consent environment. Wales has developed a ‘soft’ presumed consent approach which attempts to bridge the difficult policy scenario of stating that relative consent is not sought but in fact, relatives are consulted about two points. First, lifestyle factors about the deceased that may impact ability to be an organ donor and second, whether the relative has any evidence as to whether he/she might think that the deceased would not have agreed to donation [40] Alternatively, countries could allow individuals to document their preferences either way in a registry even in presumed consent countries as discussed in Bilgel [34].

Another policy approach that has shown the potential for success is the priority rule model as in Singapore and Israel where individuals who have opted into organ donation will be placed higher on the organ recipient list should they need a transplant [41]. While the priority rule model may increase organ donation rates, one of the key criticisms has been that quality of donor organs may fall as those who are more likely to be sick in the future may be more inclined to register as donors. Recent work has suggested a freeze mechanism such that there is a delay between the time someone signs up to be a donor and eligibility for prioritization on the waiting list [42]. Implementing such a system is complex from a public acceptability perspective and, therefore, presently may not replace or work alongside of the current paradigm of presumed and informed consent across many countries [43].

Our study faces some important limitations stemming from both the cross sectional and self-reported nature of our data sources. Decisions and intentions in the health domain are part of a dynamic process of information uptake and circumstances surrounding decision-making including the nature of family ties [44, 45]. Our cross-sectional and self-reported data captures a single moment in time for respondents and countries but nonetheless provides us a robust depiction of that moment. Another limitation to our study is that organ donation desires may not be the same for all organs [5, 6].

Nevertheless, our study makes an important contribution to the literature. We have now presented evidence suggesting that the importance of legislative environment is moderated by the role of the family in vetoing donation. Routine family consent policies attenuate the effect of presumed consent legislation, and are associated with reduced willingness to donate a relative’s organs.

Together with attitudinal findings, this suggests the need to involve the family in actual donation decisions from the beginning to avoid vetoing behaviour, engage in active efforts to work with patients’ families to enhance donation levels and/or encourage advance directives that cannot be overruled by relatives.

Encouraging advance directives and living wills to settle any family disputes about donation and remove ambiguity about decision-making [26] should be a key policy instrument to ensure that donation wishes (either allowing or not allowing) are followed. Crystallizing individuals’ wishes prior to death through pre-commitments would perhaps be even more instrumental in encouraging donation rates than policy alone.

## Electronic supplementary material

Below is the link to the electronic supplementary material.

Supplementary file1 (DOCX 112 kb)
