# Supplementary material for: ‘Relative Consent’ or ‘Presumed Consent’? Organ donation attitudes and behaviour
Source: Eur J Health Econ. 2020 Jul 10;22(1):5–16. doi: 10.1007/s10198-020-01214-8 (PMC7822792; doi:10.1007/s10198-020-01214-8)
Supplement: Supplementary file 1 — Supplementary file1 (DOCX 112 kb) [file 10198_2020_1214_MOESM1_ESM.docx]

Supplementary Materials:

Figure S1

Tables S1-S2


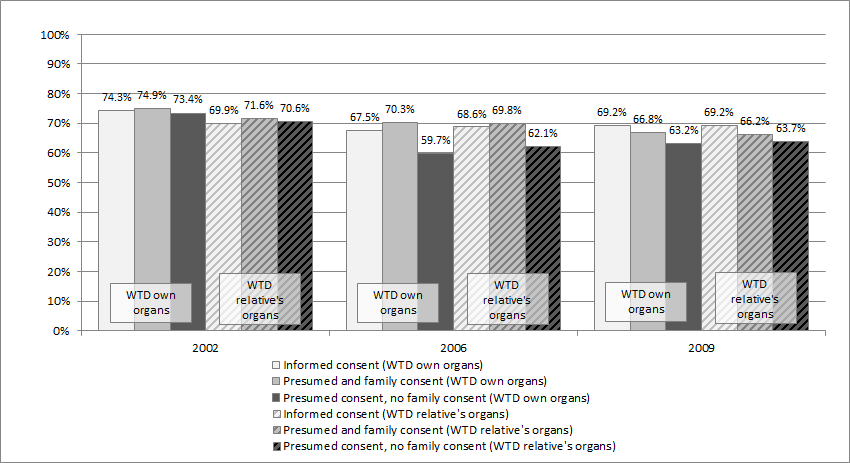


**Fig. S1: Average WTD own and relative’s organs in countries without presumed consent; countries with presumed consent only; and countries with presumed consent and routine family consent, 2002- 2009**

Informed consent countries Denmark, Germany, Ireland, Lithuania, Netherlands, Romania, United Kingdom. Presumed consent without family consent countries being Austria, Czech Republic, Latvia, Luxembourg, Poland, Sweden, Presumed and family consent: Belgium, Bulgaria, Croatia, Cyprus, Estonia, Finland, France, Greece, Hungary, Italy, Portugal, Slovak Republic, Slovenia, Spain, Turkey. Values for 2002 only include 15 EU member countries before 2004.

Source: Eurobarometer 58.2, 66.2 and 72.3 from 2002, 2006 and 2009

**Table S1: Robustness checks for regression models predicting individuals’ WTD own and family member’s organs**

|  | **WTD own organs** | | | **WTD family member’s organs** | | |
| --- | --- | --- | --- | --- | --- | --- |
| VARIABLES | **Model (3)** | **Model (3) logit** | **Model (3) Including don’t know answers †** | **Model (3)** | **Model (3) logit** | **Model (3) Including don’t know answers †** |
| **Regulation covariates** |  |  |  |  |  |  |
| *Donation policy (reference category: informed consent)* |  |  |  |  |  |  |
| Presumed consent, no family consent | 0.154** (0.074) | 0.156** (0.025) | 0.129 (0.079) | 0.179* (0.098) | 0.179* (0.100) | 0.230*** (0.069) |
| Presumed and family consent | 0.076 (0.051) | 0.076 (0.050) | 0.128** (0.057) | 0.049 (0.064) | 0.049 (0.065) | 0.099* (0.052) |
| **Family covariates** |  |  |  |  |  |  |
| Organ donation discussed | 0.274*** (0.015) | 0.271*** (0.017) | 0.308*** (0.022) | 0.238*** (0.011) | 0.236*** (0.013) | 0.244*** (0.011) |
| **Interaction regulation/family covariates** |  |  |  |  |  |  |
| Interaction organ donation discussed * presumed consent (no family consent) | -0.007 (0.029) | 0.002 (0.029) | -0.020 (0.030) | -0.004 (0.024) | 0.005 (0.024) | -0.009 (0.019) |
| Interaction organ donation discussed * presumed consent * family consent | -0.012 (0.023) | -0.007 (0.025) | -0.004 (0.027) | 0.007 (0.022) | 0.010 (0.023) | 0.026 (0.020) |
| Controls | Yes | Yes | Yes | Yes | Yes | Yes |
| Country-wave fixed effects | Yes | Yes | Yes | Yes | Yes | Yes |
| ***Model statistics*** |  |  |  |  |  |  |
| Observations | 49,718 | 49,718 | 70,013 | 49,718 | 49,718 | 70,013 |
| Pseudo R-squared | 0.156 | 0.156 | 0.131 | 0.127 | 0.127 | 0.095 |

*** significant at the 1% level; ** 5% level. SE shown in parentheses.
Marginal effects from probit specification unless stated otherwise.
**†** Instead of excluding don’t know answers, this robustness check includes ‘don’t knows’ recoded to “not willing to donate”.

**Table S2: Robustness checks for regression models predicting log of cadaveric donation rates, 2001-2010**

|  | (1) | (2) | (3) | (4) | (5) |
| --- | --- | --- | --- | --- | --- |
| VARIABLES | Full sample (28 countries) | EU-15 sample (15 countries) | Abadie & Gay sample (17 countries) **†** | Western Christian sample (24 countries) **‡** | One year - 2010 (28 countries) |
|  |  |  |  |  |  |
| **Regulation covariates** |  |  |  |  |  |
| *Donation policy (reference category: informed consent)* |  |  |  |  |  |
| Presumed consent, no family consent | 0.433 | -0.675 | 0.0361 | -0.602 | 0.313 |
|  | (1.245) | (0.541) | (0.0505) | (0.601) | (0.614) |
| Presumed and family consent | 0.369 | -0.464 | 0.368* | -0.261 | -0.0931 |
|  | (0.410) | (0.599) | (0.213) | (0.613) | (0.450) |
| **Family covariates** |  |  |  |  |  |
| Organ donation discussed by >50% of population | -0.317** | -0.330** | -0.297** | -0.301* | -0.462 |
|  | (0.159) | (0.161) | (0.145) | (0.161) | (0.507) |
| **Interaction regulation/family covariates** |  |  |  |  |  |
| Presumed consent, no family consent * Majority discussed organ donation | 0.622 | 0.695 |  | 0.650 | -0.960 |
|  | (0.591) | (0.542) |  | (0.605) | (1.052) |
| Presumed and family consent * Majority discussed organ donation | 0.331** | 0.339** | 0.313** | 0.321* | 0.328 |
|  | (0.161) | (0.159) | (0.152) | (0.166) | (0.599) |
| **Controls** |  |  |  |  |  |
| Log GDP per capita | 0.337 | 0.0978 | 0.168 | 0.240 | 0.203 |
|  | (0.548) | (0.589) | (0.351) | (0.458) | (0.765) |
| Tertiary education attainment | 0.00825 | 0.00684 | 0.00152 | 0.00269 | 0.00971 |
|  | (0.0125) | (0.0144) | (0.00632) | (0.0122) | (0.0211) |
| Catholic country | 1.094** | 1.103** | 0.360** | 1.035* | 0.561* |
|  | (0.542) | (0.511) | (0.159) | (0.557) | (0.303) |
| Common law | 0.839 | -0.278 | -0.210 | -0.164 | -0.686 |
|  | (1.254) | (0.201) | (0.159) | (0.206) | (0.539) |
| Log motor vehicle and CV deaths | -0.268 | -0.0236 | -0.122 | -0.314 | -0.512 |
|  | (0.427) | (0.308) | (0.216) | (0.416) | (0.969) |
| Year fixed | Yes | Yes | Yes | Yes | - |
| Country effects | Yes | Yes | Yes | Yes | Yes |
|  |  |  |  |  |  |
| Observations | 210 | 148 | 150 | 184 | 28 |
| R-squared | 0.869 | 0.815 | 0.898 | 0.723 | 0.557 |

*** p<0.01, ** p<0.05, * p<0.1

**†** Following Abadie & Gay (2006): excluding Bulgaria, Croatia, Cyprus, Estonia, Greece, Latvia, Lithuania, Luxembourg, Romania, Slovak Republic, and Turkey.

**‡** Following the reasoning of Abadie & Gay for excluding some of the countries in their sample (that are not Western Christian countries). Differently to them, we keep many Eastern European countries in the sample because data that weren’t available when Abadie & Gay conducted their study have become available in the meantime. For the “Western Christian sample” we exclude Bulgaria, Cyprus, Greece, Romania, and Turkey from the full set of 28 countries.

Standard errors are bootstrapped.
